# Surgery

**Published:** 1894-06

**Authors:** C. A. Morton


### SURGERY.

There is, perhaps, no disease in which it is more important to make an early diagnosis with a view to surgical treatment than in tumours of the bladder. Diagnose an innocent papilloma early, before cystitis has set in, and remove it at once, and there is probably no more satisfactory proceeding in our surgical art. Fail to recognise it until cystitis has come on, or allow the patient to drift on month after month with cystitis, every day increasing the chances of secondary disease in the kidneys, and then when operation is undertaken it will be too late, as the disease of the kidneys will only be kindled to greater activity, and life soon ended. Hence the great importance of a recent communication by Mr. Hurry Fenwick on the diagnosis and treatment of vesical tumours,[1] the result of his observation on 100 cases. The fact that these cases were diagnosed by means of the cystoscope shows the value of this method of investigation. Mr. Fenwick says "that out of some hundreds of cases of obscure urinary disease, it has enabled me to select at least fifty cases of vesical growth, the existence of which I should have been unable to pronounce with certainty without digital exploration." But to use the cystoscope with advantage requires some practice, which few surgeons have an opportunity for, and therefore Mr. Fenwick's remarks on diagnosis by other means are of much value. It has long been well recognised that the great symptom of villous growth in the bladder was hæmaturia, and that of a special kind. The patient feels perfectly well, but he comes to us one day bringing with him a specimen of bright-red urine, about which he is very alarmed. This bleeding recurs again and again, and yet he feels perfectly well and has no pain with micturition, and no growth can be felt in the renal region. Now, if we question the patient as to how the blood comes, we shall probably find that as he begins to micturate the urine is clear, but towards the end it is mixed with blood. This is most significant of vesical growth. Only towards the end of the act, when the bladder is getting empty, does the growth get interfered with by the contraction of the bladder-wall. Gilbert Barling, in a recent paper,[2] shows how frequently hæmaturia is the first symptom in papilloma of the bladder. Out of 76 cases, the records of which he has collected, it was present in 73, and

[1] *Brit. M. J.*, 1893, vol. i., p. 1209.
[2] *Birmingh. M. Rev.*, vol. xxxii., 1892, p. 205.

was the first symptom in 62. It has generally been considered that hæmaturia without pain or increased frequency of micturition indicates an innocent papilloma. Sir Henry Thompson considers that in malignant tumours the bleeding was almost invariably preceded by pain or "irritation of the bladder." Barling however shows that out of 86 cases of malignant growth, hemorrhage was the first symptom mentioned in 58;[1] and Fenwick believes that the softer forms of villous epithelioma may cause hæmaturia as the only symptom for months, and that often of a very profuse character.[2] Mr. Reginald Harrison[3] also states that papilloma in the bladder as elsewhere is liable to become cancerous and invade the bladder-wall, and he refers to a case of Mr. Paul's, in which a papilloma was removed several times in ten years and then caused death, when it was found infiltrating the bladder-wall.

Fenwick[4] calls attention to the fact that fragments of villous processes discovered in the urine (which he considers can be done in 19 per cent.) do not by any means indicate the innocent nature of the growth, for "41 per cent. of vesical carcinoma have a surface covering of villous processes." He considers that attempts should not be made to extract such fragments by sounding or scraping for diagnostic purposes, as hemorrhage is liable to result, which may be very troublesome, or cystitis may be set up. Even the plan recommended by Sir H. Thompson of using the evacuating litholapaxy syringe and catheter to withdraw fragments may cause considerable hemorrhage. Mr. David Wallace points out[5] that in these cases hæmaturia is sometimes due to detachment of a villous process, and hence such a procedure would be likely to start very considerable bleeding. In one of Mr. Wallace's cases the detachment and passage of a portion of the growth was always accompanied by severe hemorrhage.

Fenwick considers the welfare of the patient largely depends on the careful prevention of the onset of cystitis. Once let cystitis start and our chances of successful removal by operation are materially lessened. If the bladder has to be washed out for hemorrhage or retention from the presence of clot, the utmost care should be taken to have the instrument aseptic and the fluid sterile.

There is one condition which is generally considered a contra-indication to any attempt at removal of a vesical growth, and that is infiltration of the wall of the bladder as discovered by examination from the rectum or vagina, which indicates its malignant nature. But at the meeting of the Clinical Society on March 30th of this year,[6] a case was reported in which a malignant growth, which had recurred twice, was removed by

1 *Loc. cit.* 2 *Loc. cit.*
3 *Lectures on Surgical Diseases of the Urinary Organs*, 1893, p. 501.
4 *Loc. cit.* 5 *Edinb. M. J.*, vol. xxxviii., part ii., 1893, p. 731.
6 *Brit. M. J.*, 1894, vol. i., p. 752.

the suprapubic method, together with the portion of the bladder-wall from which it grew. Mr. Fenwick did not advise the operation in cases in which the peritoneum would have to be opened. Although tumours of the bladder are very often situated at the base, and therefore are uncovered by peritoneum, yet unfortunately they are often placed close to the orifice of a ureter, and this could hardly be removed. There have been a few cases recorded within the last few years of removal of a portion of the fundus of the bladder for malignant growth. Some of these are reported by continental surgeons, and one by Bruce Clarke in this country. In one case the peritoneum was peeled off the growth; in another it was removed with it; and even more extensive operations, such as altering the course of the ureters and then freely resecting the bladder, have been proposed by some London surgeons. An account of resection of the bladder-wall for carcinoma will be found in a paper by Gilbert Barling in the *Birmingham Medical Review* for March, 1890.

I operated last summer on a case of innocent villous tumour of the bladder, which well illustrates the need for early diagnosis of such cases with a view to treatment. There was a history of eight years' hæmaturia and occasional attacks of pain with micturition. On closely questioning the patient she told us that at times, not always, the blood had only come at the end of micturition. She was in great misery with the frequency and pain of micturition, and at night, even after a hypodermic injection of morphine, could get no rest. Having examined the urethra, and satisfied myself there was no urethral growth, under ether, I digitally explored the bladder. At first I felt nothing, and then I just touched what felt like blood-clot, so soft was it, floating against my finger. A minute portion was removed with forceps and spread out in water, and its papillomatous nature was at once apparent. The next day I removed a very large papilloma growing from the base of the bladder by the supra-pubic method; but it was too late, the urine was already ammoniacal and full of muco-pus, and she died in three days, with constant vomiting, probably from surgical kidneys. She had been seen by several medical men during the eight years, and had been sounded, but no digital exploration of the bladder had been made.

I have already referred to the use of the cystoscope in the diagnosis of vesical growths. Mr. Wallace,[1] whilst recognising the difficulty of the proceeding in certain cases considers that just as it is the rule to find the stone by sounding before operation, so it ought to be the custom to find the tumour with the cystoscope before attempting its removal. He admits that the appearance of the growth will not always determine its innocent or malignant nature, but that is so often with growths in other situations. Albarran says: "If the tumour be villous, with a thin pedicle, it is probably benign; while if the villi spring from

[1] *Loc. cit.*

a more solid surface—*i.e.*, seem to be implanted—the tumour is more probably malignant." Barling[1] also speaks well of the value of the cystoscope in the diagnosis of bladder tumours. He says "it leaves little to be desired, but in some cases the constant presence of blood in large quantities prevents a successful inspection with the instrument." The use of the cystoscope also might very readily induce the hæmaturia, when not already present. In writing about the differential diagnosis of cases of malignant disease of the kidney from bladder tumour, he says that in the former the use of the cystoscope will generally prove conclusively that the bleeding does not come from the bladder. But can we trust to negative evidence from cystoscopic examination? Our field of vision at one time is a very limited one, and in the frequent movement of the light from place to place we might easily miss a small growth.

How readily we may be misled by the use of the cystoscope (which requires practice just as the laryngoscope and ophthalmoscope do) is shown by reference to the beautiful atlas of cystoscopy just issued by Burckhardt and Fenwick. Folds of mucous membrane might easily be mistaken for a new growth, and distended glands in the mucous membrane of a healthy bladder might cause serious error in diagnosis. Villous growths happily seem unmistakable in the plates. Dr. Otis K. Newell, under the title of "A New and Positive Method of Bladder Examination,"[2] describes the aid to cystoscopic examination of bladder tumours derived from pushing up the base of the bladder with the finger in the rectum. The best account of cystoscopy is Hurry Fenwick's book on the subject[3] (not the atlas just referred to), in which he says that the old platinum loop cystoscope of 1879 has the same relation to that of 1887 as the "Puffing Billy" of Stephenson to later locomotives.

Watson[4] recognises the great difficulty there may be in getting a clear view of the bladder in cases of hæmaturia, but he says that "except when it is abundant and constant, a satisfactory view with the cystoscope can usually be obtained during a period of intermission of the bleedings, and if a moderate bleeding be excited by the introduction of the tube, it can usually be checked by prolonged irrigation with cold water, or, this failing, with very hot water."

In some cases a greatly enlarged prostate makes a cystoscopic examination in the ordinary way impossible. Hurry Fenwick in such cases proposes[5] to pass the instrument into the bladder through a cannula inserted above the pubes, so that the prostatic pouch could be thoroughly explored. If a growth were discovered the cannula opening could be enlarged and the growth removed. This proceeding, Mr. Fenwick points out, could replace supra-pubic exploratory cystotomy.

[1] *Loc. cit.* [2] *Med. Rec.*, vol. xliii., 1893, p. 261.
[3] *Electric Illumination of the Bladder and Urethra*, 1889.
[4] *Internat. Clin.*, vol. iii., 1892, p. 180.
[5] *Brit. M. J.*, 1894, vol. i., p. 860.

Nicoll[1] has lately pointed out some of the difficulties in the use of the cystoscope, but considers it an instrument of the greatest practical value in surgery.

Barling[2] describes a bimanual method of examination for vesical growths, which has only recently, he thinks, received the attention it deserves. We can understand its value in cases of carcinoma (and more especially in sarcoma, where usually the growth is larger), but we should hardly expect to determine the presence of an innocent growth composed entirely of delicate villous processes even if it had a fairly firm pedicle.

Formerly, if in some cases of intractable cystitis or recurrent hæmaturia, we were in doubt whether a tumour were not the cause, Sir Henry Thompson recommended a digital exploration through the perineum. Cystoscopy may now replace this operation in many cases: but there remain some in which either perineal or supra-pubic incision into the bladder is of great value not only for diagnosis but also for drainage. Reginald Harrison writes[3] with reference to such a proceeding in cases of chronic cystitis with offensive urine, in which there is no stone or vesical growth. He has thus operated in ten cases "with results which have proved uniformly successful and free from risk of any kind." In the case which Mr. Harrison relates in detail, he found the bladder much pouched in two places, and thinks the urine retained in these pouches near the rectum was the cause of the offensive urine and cystitis. The patient recovered after supra-pubic cystotomy and thorough flushing. I have lately had under my care a man 55 years of age (without prostatic enlargement), who came into the Bristol General Hospital, after months of painful micturition, with his urine almost solid with muco-pus. There was no history of hæmaturia at any time, and sounding detected no stone, nor did digital examination discover any infiltration at the base of the bladder. Washing out the bladder and leaving in iodoform emulsion greatly improved the condition of the urine. Had it not done so I intended at once to drain and explore the bladder by a supra pubic opening. He then got an attack of continuously high temperature, 102°–103° for a week, without fresh symptoms of any kind; but we detected distinct bilateral renal tenderness. After this he became more and more feeble, and died. There was found intense cystitis, with a pouch on the posterior aspect of the bladder, about the size of a walnut, with very thin walls. The bladder was contracted somewhat, but not greatly thickened or fasciculated. There was double pyelitis with suppurative nephritis, the onset of which had probably caused the high temperature. This we suspected to be the case during life, and, in addition to his extreme weakness, prevented any operation for drainage as performed by Mr.

[1] *Glasgow M. J.*, vol. xl., 1893, p. 418.

[2] *Birmingh. M. Rev.*, vol. xxxii., 1892, p. 205.

[3] *Med. Week*, vol. ii., 1894, p. 133.

Harrison. Had we been able to operate earlier, before suppurative nephritis was set up, life might have been saved, and could I have foreseen the onset of the nephritis so soon after admission I would not have been content to wait even though the urine was so markedly improving in character.

Mr. Targett has recently described vesical sacculi very fully in a paper on "The Pathology of Cystic Tumours connected with the Bladder."[1]

C. A. Morton.

### OBSTETRICS.

[1] *Brit. M. J.*, 1893, vol. ii., p. 218.

